# Sex Specific Differences in Response to Calorie Restriction in Skeletal Muscle of Young Rats

**DOI:** 10.3390/nu14214535

**Published:** 2022-10-28

**Authors:** Margalida Torrens-Mas, Cayetano Navas-Enamorado, Devin Wahl, Andres Sanchez-Polo, Anna Picca, Jordi Oliver, Pilar Roca, Marta Gonzalez-Freire

**Affiliations:** 1 Translational Research in Aging and Longevity (TRIAL) Group, Health Research Institute of the Balearic Islands (IdISBa), 07120 Palma de Mallorca, Spain; 2 Department of Health & Exercise Science, Center for Healthy Aging, Colorado State University, Fort Collins, CO 80521, USA; 3 Fondazione Policlinico Universitario “A. Gemelli”, IRCCS, 00168 Roma, Italy; 4 Department of Medicine and Surgery, LUM University, 70010 Casamassima, Italy; 5 Grupo Multidisciplinar de Oncología Traslacional, Institut Universitari d’Investigació en Ciències de la Salut (IUNICS), Universitat de les Illes Balears, 07122 Palma de Mallorca, Spain

**Keywords:** calorie restriction, aging, mitochondria, inflammation, autophagy, senescence

## Abstract

Calorie restriction (CR), defined as a reduction of the total calorie intake of 30% to 60% without malnutrition, is the only nutritional strategy that has been shown to extend lifespan, prevent or delay the onset of age-associated diseases, and delay the functional decline in a wide range of species. However, little is known about the effects of CR when started early in life. We sought to analyze the effects of CR in the skeletal muscle of young Wistar rats. For this, 3-month-old male and female rats were subjected to 40% CR or fed ad libitum for 3 months. Gastrocnemius muscles were used to extract RNA and total protein. Western blot and RT-qPCR were performed to evaluate the expression of key markers/pathways modulated by CR and affected by aging. CR decreased body and skeletal muscle weight in both sexes. No differences were found in most senescence, antioxidant, and nutrient sensing pathways analyzed. However, we found a sexual dimorphism in markers of oxidative stress, inflammation, apoptosis, and mitochondrial function in response to CR. Our data show that young female rats treated with CR exhibit similar expression patterns of key genes/pathways associated with healthy aging when compared to old animals treated with CR, while in male rats these effects are reduced. Additional studies are needed to understand how early or later life CR exerts positive effects on healthspan and lifespan.

## 1. Introduction

Calorie restriction (CR), defined as a reduction of the total calorie intake by 30% to 60% without malnutrition, has been shown to extend lifespan across a wide range of species [1,2]. Furthermore, CR has been proposed as a robust strategy to delay the functional decline associated with aging thereby preventing the onset of most age-related diseases [1,3]. Several conserved signaling pathways are modulated by CR, including the mechanistic target of rapamycin (mTOR) pathway, the insulin-like growth factor (IGF)-insulin pathway, the AMP-activated protein kinase (AMPK) pathway, the nuclear factor erythroid 2-related factor 2 (NFE2L2/NRF2) pathway, or via the modulation of Sirtuins [3,4].

CR is thought to exert a protective role on muscle aging, as it preserves muscle mass and function [5]. Although some reports showed no differences in mitochondrial biogenesis, mitochondrial function was found to be preserved in skeletal muscle of animals under CR [6,7]. Furthermore, CR has been shown to improve proteostasis in skeletal muscle, as it modulates protein degradation to prevent the accumulation of oxidative-damaged proteins [8,9]. The attenuation of oxidative stress damage has also been reported in rat cardiac muscle, as well as activation of autophagy with CR [10]. In addition to a reduction in oxidative stress, inflammation levels have also been found reduced in the skeletal muscles of animals under CR [9,11].

However, little is known about the effects of CR when started early in life. Some studies have reported no differences in mitochondrial function or antioxidant enzymatic activities [12], in oxidative metabolism [13], or in mTOR signaling and protein degradation [14] in young rats subjected to CR. Therefore, the aim of this study was to analyze the effects of CR in the skeletal muscle of young Wistar rats. For this, male and female rats aged 3 months were subjected to 40% CR or fed ad libitum for 3 months. Following treatment, gastrocnemius muscles were used to determine the levels of markers of mitochondrial function, antioxidant, autophagy, nutrient-sensing, inflammation, and senescence pathways.

## 2. Materials and Methods

### 2.1. Animals and Diets

All experiments were performed according to general guidelines for animal care approved by the institutional ethics committee and EU regulations (2010/63/UE). Female and male Wistar rats aged 3 months were purchased from Charles River Laboratories (Barcelona, Spain) and housed at 22 °C and 65 ± 3% humidity with a 12-h light/dark cycle. Animals were divided into four experimental groups with different diets for 12 weeks. One group of females (*n* = 8) and one group of males (*n* = 3) were fed ad libitum with a pelleted standard diet (A04, Panlab, Barcelona). Another group of females (*n* = 8) and of males (*n* = 4) were subjected to 40% CR. Food for CR treated-rats consisted of the same standard diet but was adjusted weekly and dependent on the average food consumed by the same sex ad libitum group, which was used as a reference for baseline food consumption. This was done to correct for growth requirements and avoid delays in development.

At 24 weeks of age, rats were sacrificed by decapitation at the beginning of the light cycle and tissues were collected, weighed, immediately frozen in liquid nitrogen and stored at −80 °C until further analyses. Serum glucose and triglycerides were measured using an Accutrend^®^ GCT-meter (Roche Diagnostics, Basel, Switzerland). 

### 2.2. Total Protein Extraction

Gastrocnemius muscles (50 mg) were placed in a chilled mortar and pulverized with a pestle using liquid nitrogen. RIPA buffer (50 mM Tris-HCl pH 7.5, 150 mM NaCl, 0.1% SDS, 0.5% deoxycholate, 1% Triton X-100, 1 mM EDTA) with protease and phosphatase inhibitors (Halt™ protease and phosphatase inhibitor Single-use cocktail, EDTA-free, 100x, ThermoFisher Scientific, Madrid, Spain, #78443) was added (10% *w*/*v*). Samples were sonicated on ice at 40% amplitude for 10 s three times (VibraCell 75185, VWR, Barcelona, Spain). Then, samples were centrifuged at 600× *g* for 10 min at 4 °C to remove any debris. Supernatants were recovered and protein content was quantified using the bicinchoninic acid (BCA) protein assay kit (Pierce, Germany). 

### 2.3. Western Blot Analysis

For each blot, 40 µg of total protein was separated on SDS-PAGE gels and electrotransferred to 0.22 µm nitrocellulose membranes using the Transblot Turbo transfer system (Bio-Rad). Ponceau S staining was used as a loading control. The intensity of each lane was measured, and the quantification of each protein was corrected to the total amount of protein loaded for each sample. Membranes were then blocked with 5% non-fat powdered milk in Tris Buffer Saline, pH 7.6 (TBS) with 0.05% Tween-20 for 1 h. Antibodies against proteins analyzed are listed in Supplemental Table S1. After overnight incubation with primary antibodies, membranes were incubated with secondary antibodies conjugated with horseradish peroxidase for 1 h and protein bands were detected with Immun-Star™ WesternC™ Chemiluminiscent Kit (Bio-Rad) Western. The chemiluminiscence signal was acquired with a Chemidoc XRS densitometer (Bio-Rad) and results were analyzed with Quantity One Software (Bio-Rad).

### 2.4. Determination of ATPase Enzymatic Activity

Gastrocnemius muscles were homogenized in sucrose buffer (40 mM sucrose, 220 mM mannitol, 1 mM EDTA, 10 mM Tris-HCl, pH 7.4) with a Teflon/glass homogenizer. Samples were then centrifuged at 600× *g* for 10 min at 4 °C and supernatants were recovered. Protein content was analyzed with a Bradford assay and the enzymatic activity of Complex V (ATPase, EC 3.6.1.3) was performed immediately after. Briefly, samples were incubated in a 96-well spectrophotometric plate with the assay buffer (0.33 M sucrose, 6.3 mM MgSO_4_, 63.66 mM HEPES, 0.442 mM NADH, pH 8.0) with 2.5 mM phosphor(enol)pyruvic acid, 0.5 µg/mL Pyruvate Kinase, 0.25 µg/mL L-Lactic dehydrogenase and 0.1 µg/mL antimycin. To start the reaction, 5 mM ATP was added into each well. Oxidation of NADH at 340 nm was followed for 15 min at 37 °C. The generated slope is inversely proportional to the activity of Complex V. The enzymatic activity was expressed as international units (I.U.) per mg of protein, with an extinction coefficient used of 6.22 mM^−1^ cm^−1^.

### 2.5. RT-qPCR

Gastrocnemius muscles (25 mg) were pulverized in liquid nitrogen as previously described and 1 mL of Tri Reagent^®^ (Sigma-Aldrich, St. Louis, Missouri, United States) was added to each sample. RNA was isolated following manufacturer’s instructions and quantified using a BioSpec-nano spectrophotometer (Shimadzu Biotech, Kyoto, Japan). 

Total RNA (1 µg) was reverse transcribed to cDNA at 37 °C for 50 min with 200 U M-Mlv reverse transcriptase in a 20 µL volume of reaction mixture containing 1X First-Strand Buffer, 2.5 µM random hexamers, 500 µM each dNTP, 20 U RNAse inhibitor, and 10 mM DTT. Samples were diluted 1/10 and stored at −20 °C until use. 

PCR reactions were performed using SYBR Green chemistry on a LightCycler^®^ 480 System (Roche, Barcelona, Spain). Target genes, primers, and annealing temperatures are specified in Supplemental Table S2. The reaction mixture contained 7.5 µL SYBR TB Green^®^ Premix Ex Taq™ (RR420A, Takara, Shiga, Japan) with 0.5 µM of forward and reverse primers) and 2.5 µL of cDNA. The amplification program included a denaturation step at 95 °C, 5 min, followed by 45 cycles with a denaturation step (10 s, 95 °C), an annealing step (10 s, temperature depending on primers, see Supplemental Table S2), and an elongation step (12 s, 72 °C). A negative control without cDNA was run in each assay. Ct values obtained were analyzed, considering the efficiency of each reaction, using the GenEx Standard Software (Multi-DAnalises, Göteborg, Sweden). Two housekeeping genes, *Rpl32* and *Tbp*, were used according to the Normfinder algorithm. 

### 2.6. String Analysis

Protein–protein interactions (PPIs) were analyzed using the STRING database (v.11.5; www.string-db.org, accessed on 24 August 2022) by querying genes that were differentially expressed genes and proteins obtained in the results. The network edges represent either the evidence of the PPI or the confidence. All active interaction sources were selected, and the minimum required interaction score was set at medium confidence (0.400). 

### 2.7. Statistical Analysis

Results are presented as mean ± Standard error of the mean (SEM) unless otherwise specified. Differences between CR and control groups were assessed by Student’s *t*-test, and interaction effects between sexes were generated by two-way analysis of variance (ANOVA) and post hoc Tukey’s test. A *p* value < 0.05 was considered statistically significant. To test potential associations between the measured parameters, a principal component analysis (PCA) was computed. Statistical analyses were performed using R Studio version 3.5.2 of the R programming language (R Project for Statistical Computing; R Foundation, Vienna, Austria). 

## 3. Results

### 3.1. CR Reduced Body and Tissue Weights

**Figure 1:**
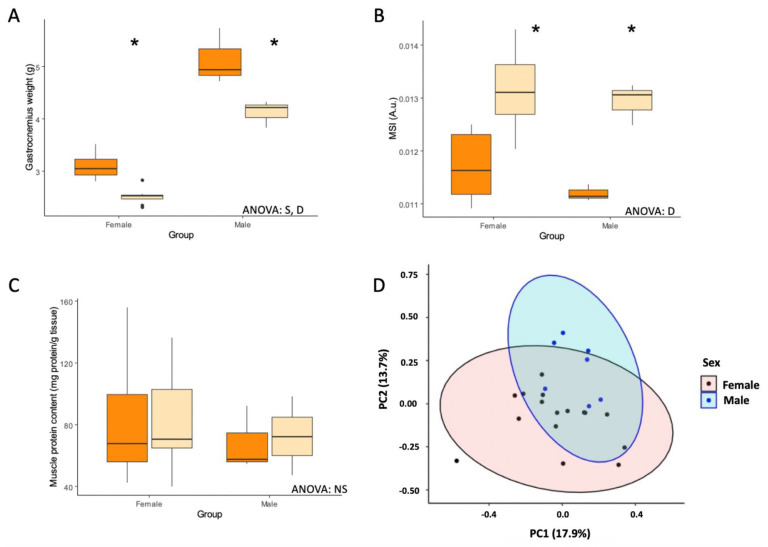
Effect of calorie restriction on skeletal muscle. Boxplots showing (**A**) gastrocnemius weight, (**B**) muscle somatic index (MSI), and (**C**) muscle protein content in female and male rats under ad libitum or calorie restriction diets. (**D**) Principal component analysis (PCA) plot performed on all data including weights, mRNA and protein expression (excluding mitochondrial complexes). The first component explains ~18% of the variation, and the second component ~15%. Two-way ANOVA was performed to assess for significance. Abbreviations: S, sex differences; D, diet differences; N, non-significant; PC1, principal component 1; PC2: principal component 2. * Significant difference between calorie-restricted and control group.

Body mass, tissue weights, and serum levels of glucose and triglycerides of control and CR male and female rats are shown in Table 1. Body weight was significantly decreased in animals subjected to CR compared to control animals. Both sexes exhibited similar weight loss, −27.1% in females, and −31.3% in males. Treatment with CR also reduced the weight of adipose tissue (i.e., inguinal, lumbar, mesenteric, and ovarian/epididymal adipose tissues) and the liver (−62.2% and −31.3% in females, and −67.0% and −33.2% in males, respectively). Skeletal muscle weight was lower in female rats compared to their male counterparts and CR reduced muscle weight similarly in both sexes. However, the muscle somatic index (MSI), obtained by adjusting muscle skeletal weight to body mass, was increased in both male and female CR rats, suggesting that skeletal muscle specifically is preserved with CR in young animals. In fact, total protein content of skeletal muscle tissue remained unchanged with diet (Figure 1A–C). Finally, serum glucose was unaffected by diet, while triglycerides were reduced by 50% but this effect was seen only in male rats subjected to CR. Finally, principal component analysis (PCA) showed a clear separation between female and male rats treated with CR (Figure 1D).

### 3.2. CR Effects on Mitochondrial Function and Dynamics

**Figure 2:**
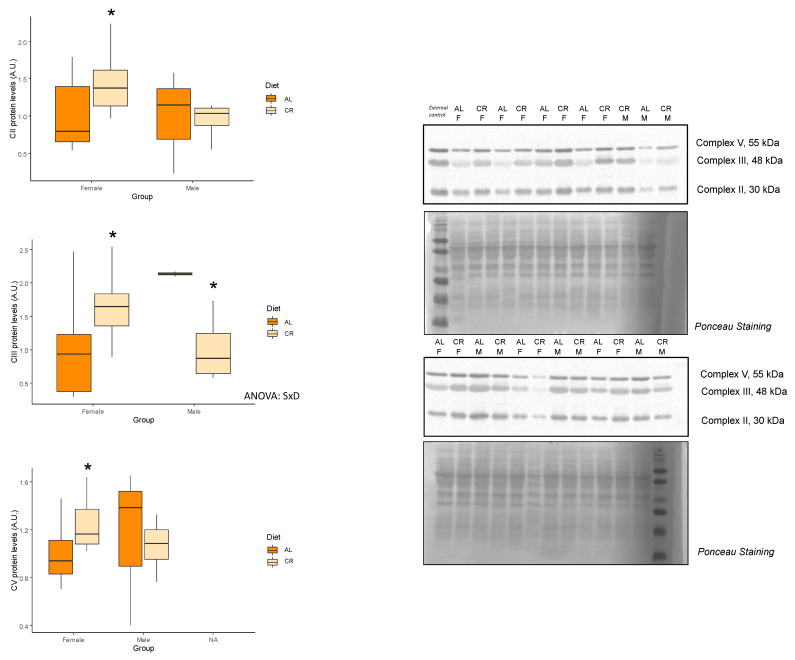
Effects of calorie restriction on oxidative phosphorylation (OXPHOS) complexes. Boxplots showing the protein expression levels of Complex II, Complex III, and Complex V and representative bands of the Western blot. Two-way ANOVA was performed to assess for significance. Abbreviations: SxD, interactive effect between sex and diet; D, diet differences; NS, non-significant; AL: ad libitum; CR: caloric restriction; F: female; M: male. * Significant difference between calorie-restricted and control group.

**Figure 3:**
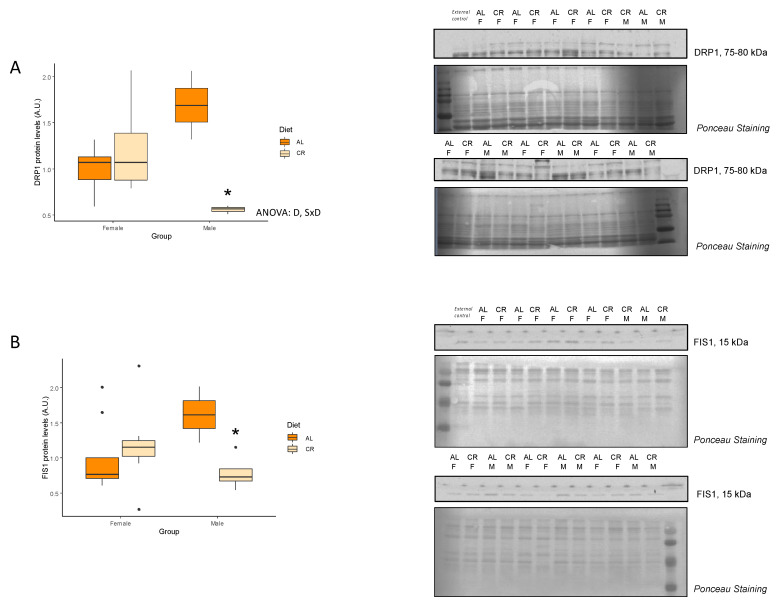
Effects of calorie restriction on proteins related to mitochondrial dynamics. Boxplots showing the protein levels of (**A**) DRP1 and (**B**) FIS1, and representative bands of the Western blot. Two-way ANOVA was performed to assess for significance. Abbreviations: SxD, interactive effect between sex and diet; D, diet differences; NS, non-significant; AL: ad libitum; CR: caloric restriction; F: female; M: male. * Significant difference between calorie-restricted and control group.

To evaluate the effects of early onset CR on mitochondrial bioenergetics in skeletal muscle, protein levels of the oxidative phosphorylation (OXPHOS) complexes were analyzed by Western Blot (Table 2, Figure 2), as proteins involved in the main bioenergetic function of these organelles. Complex I and V showed no differences with CR regardless of the sex, while complexes II, III, and V were increased in CR but only female rats. On the other hand, complex III was decreased with CR, but this effect was seen only in male rats. Importantly, Complex IV/Complex V ratio remained unvaried with CR in both sexes. Mitochondrial dynamics were also evaluated via the analysis of mitochondrial fission-related proteins GTPase dynamin-related protein 1 (DRP1) and mitochondrial fission 1 protein (FIS1) levels. Interestingly, there were not any differences in levels of these proteins in female rats subjected to CR, but male rates undergoing CR exhibited significant decreases in both DRP1 and FIS1 expression levels (Figure 3).

Complex V or ATPase enzymatic activity was measured to obtain an indirect measure of skeletal muscle function. ATPase activity remained unchanged with CR in both sexes (*p*-value = 0.429) (Figure S1). We observed a significant sex difference in ATPase activity (*p*-value < 0.01), with females showing twice the activity as males. 

### 3.3. Antioxidant Proteins

**Figure 4:**
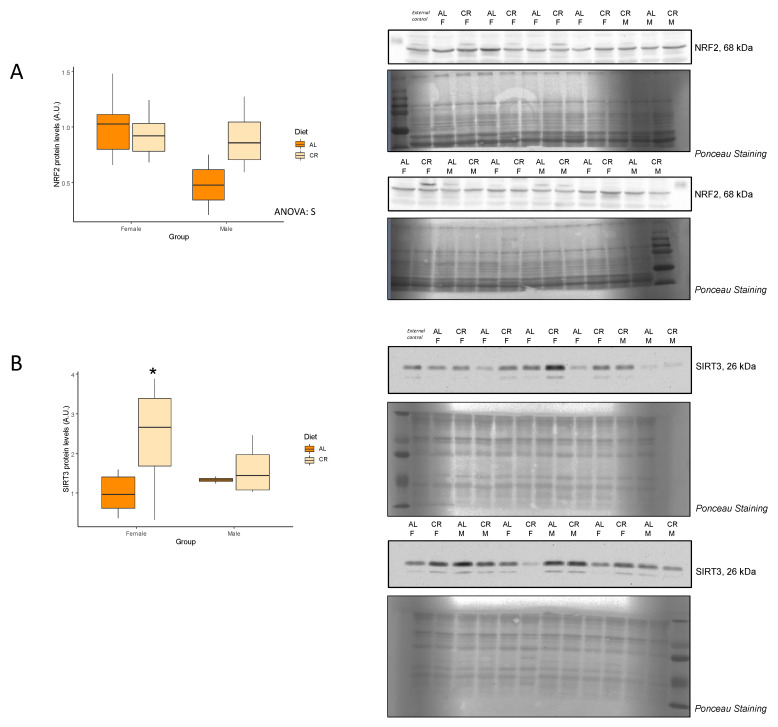
Protein levels of (**A**) NRF2 and (**B**) SIRT3 antioxidant factors. Boxplot showing the protein levels of NRF2 and SIRT3, and representative bands of the Western blot. Two-way ANOVA was performed to assess for significance. Abbreviations: S, sex differences; AL: ad libitum; CR: caloric restriction; F: female; M: male. * Significant difference between calorie-restricted and control group.

Table 3 shows the mRNA levels and Table 4 the protein levels of several key antioxidant proteins that are involved in aging and modulated by CR in older age. Interestingly, in younger rats, we were not able to detect differences in most of these genes/proteins but there was an increase in the level of sirtuin 3 (SIRT3) protein in female CR rats (Figure 4). Interestingly, no changes were observed in acetylated levels of superoxide dismutase 2 (SOD2), a known target of SIRT3. On the other hand, NRF2 expression levels, a key transcription factor involved in antioxidant pathways, were different between males and females but not influenced by CR (Figure 4).

### 3.4. Autophagy and Apoptosis

**Figure 5:**
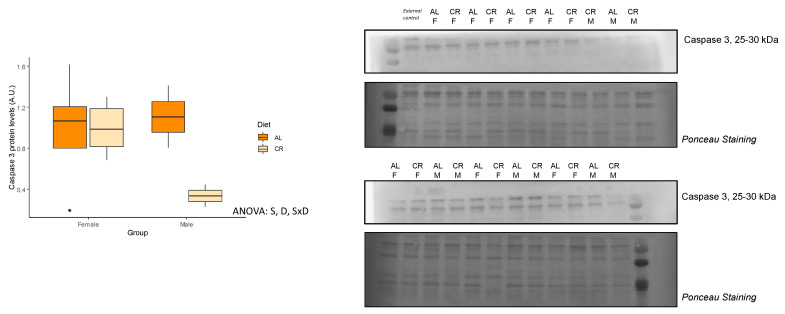
Protein levels of caspase 3. Boxplot showing the protein levels of Caspase 3 and representative bands of the Western blot. Two-way ANOVA was performed to assess for significance. Abbreviations: S, sex differences; D, diet differences; SxD indicates interactive effect between sex and diet; AL: ad libitum; CR: caloric restriction; F: female; M: male.

mRNA levels of *Map1lc3a* and *Sqstm1* (Table 5), and protein levels of LC3 and Caspase 3 (Table 6) were measured as key makers of autophagy and apoptosis. No differences among groups were observed in *Map1lc3a* and *Sqstm1* gene expression, and no changes were found in LC3A/B protein levels or the LC3-II/LC3-I ratio. However, we did detect interactive effects between sex and diet for caspase 3 and noted significant decreases in caspase 3 protein expression levels, but only in male rats subjected to CR (Figure 5).

### 3.5. Nutrient-Sensing Pathways

**Figure 6:**
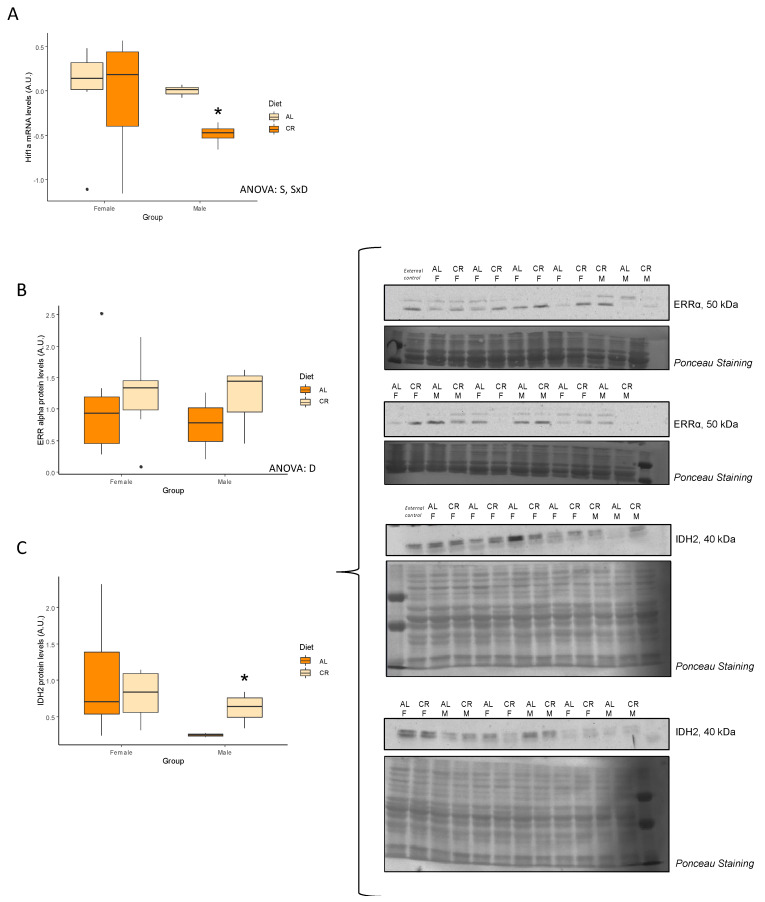
Levels of key nutrient-sensing pathways markers. Boxplots showing (**A**) the mRNA levels of *Hif1a*, (**B**) the protein levels of ERRα and (**C**) the protein levels of IDH2, and representative bands of the Western blot. Two-way ANOVA was performed to assess for significance. SxD indicates interactive effect between sex and diet, D indicates diet differences; AL: ad libitum; CR: caloric restriction; F: female; M: male. * Significant difference between calorie-restricted and control group.

Table 7 shows the mRNA levels of several key genes related to nutrient-sensing pathways. Interestingly, there were no differences between groups/sex in expression levels of *Pik3ca*, *Akt1*, *Gsk3b*, and *mTOR*. Only the expression of *Hif1a* showed an interactive effect between sex and diet and this effect was significantly reduced in male rats subjected to CR (Figure 6A).

As seen in Table 8, protein levels of insulin receptor beta (IRb) and insulin receptor substrate (IRS) were not influenced by CR or sex. Similarly, protein kinase B (AKT), liver kinase B1 (LKB1), and lactate dehydrogenase (LDH) were unaffected by diet or sex. Interestingly, only levels of estrogen-related receptor alpha (ERRα) were increased in male and female rats treated with CR (Figure 6B,C). Both AMPK and glycogen synthase kinase (GSK) showed a tendency (*p* = 0.07 and *p* = 0.05, respectively) to decrease in females rats subjected to CR. Finally, CR was associated with a decrease in isocitrate dehydrogenase 2 (IDH2) protein levels but this effect was only seen in males. 

### 3.6. Inflammation and Senescence

**Figure 7:**
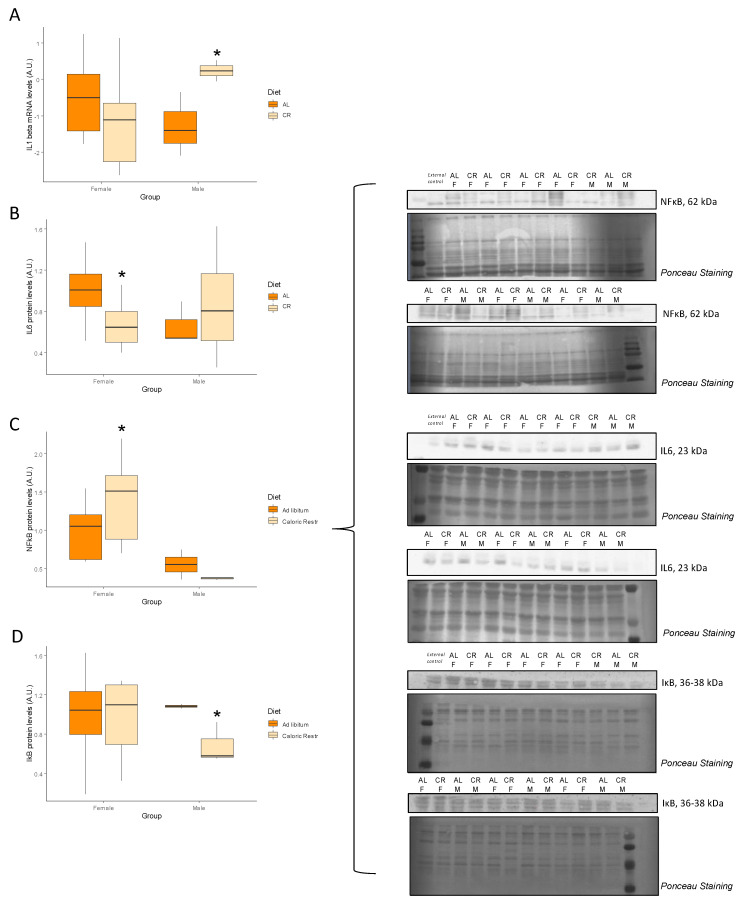
Levels of inflammation markers. Boxplots showing (**A**) the mRNA levels of *IL1B*, and the protein levels of (**B**) IL6, (**C**) NFκB, and (**D**) IκB, and representative bands of the Western blot. Abbreviations: AL: ad libitum; CR: caloric restriction; F: female; M: male. * Significant difference between calorie-restricted and control group.

Levels of key mediators of inflammation and senescence are shown in Table 9, Table 10 and Table 11. No changes were observed in mRNA levels of *Tnf* and *TGFb*, while *Il1b* (Figure 7A) expression increased but only in male rats subjected to CR, while interleukin 1 receptor (IL1R) protein levels remained unvaried by diet. Interleukin 6 (IL-6), a key protein involved in inflammatory response, (Figure 7B) increased with CR only but this effect was only noted in females. On the other hand, *NF-kB* mRNA expression did not change with CR, but interestingly protein levels of nuclear factor kappa b (NF-kB) (Figure 7C) increased with CR in females. Inhibitor of nuclear factor kappa b (IkB) protein levels (Figure 7D) decreased with CR but this effect was noted only in males. We did not find any changes in *Tp53*, *Cdkn2a*, and *Sirt6* mRNA levels among groups, and only a tendency (*p* = 0.07) of increased expression of *Cdkn1a* in male rats subjected to CR. P53 protein levels were also unaffected by diet or sex. 

### 3.7. Analysis of Protein–Protein Interaction

**Figure 8:**
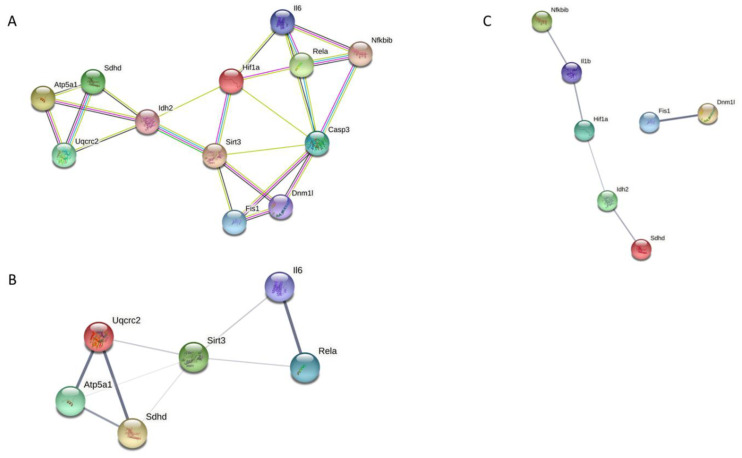
PPIs networks. (**A**) Network showing PPIs among genes and proteins differentially expressed in both sexes. (**B**) Network showing PPIs among genes and proteins differentially expressed in females. Thickness of edges represents the strength or confidence of data. (**C**) Network showing PPIs among genes and proteins differentially expressed in males. Thickness of edges represents the strength or confidence of data.

Finally, we analyzed possible protein–protein interactions using the STRING database. Figure 8A shows the main interactions and the interaction type found by querying the genes and proteins that were significantly affected by CR. The PPI enrichment *p*-value for this network was 5.05 × 10^−11^, with 24 edges. Specific networks for females and males were also constructed. In Figure 8B,C, the network with the differentially expressed genes and proteins for females and males are shown, respectively. The thickness of the edges represents the strength of data supporting each PPI. The PPI enrichment values for these networks were 0.0103 for females and 0.000826 for males. 

## 4. Discussion

In this study, we analyzed the influence of 40% CR on markers of skeletal muscle function in 3-months old male and female Wistar rats. As expected, treatment with CR resulted in a decrease in body, liver, adipose tissue, and skeletal muscle weights. We also measured the expression of some key age-related markers related to the hallmarks of aging and involved in mitochondrial biology, antioxidant activity, autophagy and apoptosis, nutrient-sensing pathways, inflammation, and senescence. Interestingly, we found significant differences among dietary treatment and sex in only some mitochondrial-related proteins and in a few key markers of antioxidant, apoptosis, inflammatory and nutrient-sensing pathways. 

In the current study, CR reduced total body weight, liver weight, fat mass, as well as muscle mass, which is in accordance with what has been previously reported [12,13,15,16]. However, MSI, an index of sarcopenia [17,18], and the total protein content of skeletal muscle, were increased with CR in both males and females. These results are agreement with previous observations in the tibialis cranialis and the soleus muscles from rats under to energy restriction [17]. Furthermore, Colom et al. [12] also reported conserved MSI and protein content in gastrocnemius muscles in male CR rats, and even an increase in protein content in female CR rats. Conversely, Miller et al. [19] showed that the synthesis of mitochondrial protein rate was similar in skeletal muscle of rats, regardless of age and CR treatment. These results, in addition to the results in the current study, indicate that skeletal muscle function might be preserved by CR in young male and female rats. 

Mitochondrial dysfunction, including alterations in the OXPHOS complexes and in mitochondrial dynamics, is as a key hallmark of aging [2,20,21]. In skeletal muscle, CR has shown to preserve mitochondrial function by increasing OXPHOS activity or mitochondrial respiration [7,12]. In the present study, we observed a slight increase in the protein level of several subunits of the oxidative phosphorylation complexes in females rats subjected to CR, although the complex IV/complex V ratio remained unvaried, thus indicating a preserved mitochondrial oxidative capacity. On the contrary, there was a decrease in the levels of complex III with CR, but this effect was only seen in male rats. Unfortunately, we could not determine the enzymatic activities of the OXPHOS complexes, which could have revealed more valuable information about mitochondrial bioenergetics. Previous studies conducted in young male rats showed no changes in OXPHOS levels or function with CR [6,22], although Wang et al. [23] suggested that the effect of CR on mitochondrial function might be fiber-type dependent. On the other hand, Gutiérrez-Casado et al. [24] reported a decrease in complex III in the skeletal muscle of mice subjected to 18 months of CR. In the current study, we observed a decrease in both DRP1 and FIS1 fission-related proteins in male rats treated with CR. Mitochondrial fission is thought to be induced in aged skeletal muscle, and long-term CR has been shown to reduce the levels of FIS1 and DRP1 [18,25]. These results suggest that fission is attenuated by CR, presumably to stimulate mitochondrial fusion or to avoid mitochondrial degradation [26]. Interestingly, in the current study, we did not observe any changes in key autophagy markers, which seem to be induced in older animals subjected to CR [24].

CR has also the potential to modulate satellite cells, the stem cells of skeletal muscle, which are involved in muscle homeostasis and regeneration [27,28]. Some reports suggest that CR increases the number of satellite cells and enhances their regenerative ability [27,29,30]. Cerletti et al. [29] showed that satellite cells from young mice under CR were metabolically reprogrammed and presented increased number of mitochondria and oxidative phosphorylation. However, Abreu et al. [30] reported that the increased proliferation of satellite cells of CR mice is not consequence of a change in metabolism.

Oxidative stress is closely related to mitochondrial dysfunction and also plays a role in aging [26]. CR has been reported to reduce oxidative damage in several tissues and animal models [7,10,25,31]. In our study, SIRT3 was increased in females under CR, although other antioxidant proteins were unvaried. This limited effect could be explained because animals were too young to ‘consider’ oxidative stress a determinant factor. In fact, previous studies have shown that the enzymatic activity of some antioxidant proteins are preserved in young skeletal muscle and these antioxidant defenses were higher in females than in males [12]. This result is consistent with our findings showing increased levels of NRF2 in females. 

Interestingly, we found that protein levels of caspase 3 was decreased in male rats under CR and was unvaried in females. Caspase 3 has been reported to be involved in muscle differentiation and the promotion of myogenesis [32,33]. In fact, this protein regulates multiple pathways involved in skeletal muscle growth. Caspase 3 is responsible for the cleavage of Pax7, a transcription factor regulating self-renewal of satellite cells [34], the stem cells of skeletal muscle. Thus, this decrease in caspase 3 would indicate a preservation of stem cell niche in males. However, other factors should be considered to further analyze the effects of CR on the satellite cell population in younger rats, including the transcription factor NF-Y [28]. On the other hand, caspase 3 also regulates p21 expression, which results in cell cycle arrest and the initiation of the cell’s differentiation program [34]. In this regard, we only observed a slight trend of increased *Cdkn1a* mRNA expression levels in males. The function of skeletal muscle was preserved in both males and females, as discussed previously and based on the MSI and total skeletal muscle protein content. 

Very few changes were observed in genes and proteins related to nutrient-sensing pathways, which is in agreement with other animal studies, suggesting that the benefits of CR are limited in younger animals whose muscle function is adequate [13,14]. In the current study, *Hif1a mRNA* expression was significantly reduced but only in males under CR. HIF1, an hypoxia-inducible factor, mediate transcriptional responses to hypoxia and has been shown to increase with aging, and CR is able to attenuate this effect [35]. On the other hand, in the current study, ERRα was increased with CR, perhaps due to its involvement in the regulation of mitochondrial function and cellular metabolism [36]. 

In our study, contradictory results were also found when analyzing the effect of sex and diet on key inflammatory markers. Male rats under CR showed increased expression of *IL1b* and decreased levels of IκB, a negative regulator of the transcription factor NF-κB [35]. These results suggest that there is an increase in inflammation when CR is started at a younger age. On the contrary to the results in males, there was a decrease in IL6 and NF-κB protein levels in younger CR treated female rats. Previous reports have shown that CR decreases key inflammatory mediators in several tissues, including skeletal muscle [11,35,37,38]. It should be noted that most of our results are indirect measures of inflammation. For instance, in the current study, only an increase in the *IL1b* mRNA expression was observed, which does not necessarily translate into higher protein levels of this cytokine. Furthermore, no downstream effects of IκB and NF-κB were observed. The decrease in IL6 levels in female CR treated rats are in agreement with results from previous studies [11,33].

Finally, we were not able to detect any changes in markers of senescence among the groups. As mentioned before, this may indicate an attenuated effect or a ceiling effect of CR in young animals, as age-related senescence is not yet a determinant factor in these animals. 

Skeletal muscle has been shown to directly modulate lifespan in several species. For instance, the overexpression of Atg8, a gene involved in autophagy, in fly muscles is sufficient to extend their lifespan [39]. CR can have an impact on skeletal muscle health, improving processes such as autophagy, proteostasis, antioxidant defenses, or mitochondrial function. Through the release of myokines and several metabolites, skeletal muscle can affect other tissues including the brain, the adipose tissue, or the liver [40]. Furthermore, avoiding the loss of skeletal muscle delays age-related diseases such as diabetes, obesity, or some types of cancer, and reduces chronic inflammation and insulin resistance [41]. Alternative to CR, maternal obesity and post-natal over nutrition have been reported to impair skeletal muscle function via glucose and lipid metabolism mechanisms, highlighting muscle metabolic dysfunction as a risk factor to develop insulin resistance and diabetes [42]. Thus, maintaining healthy, functional skeletal muscles can contribute to maintain organism homeostasis and delay aging, improving lifespan.

This study has limitations. First, the sample size was small and therefore there may be limited statistical power to detect differences in gene or protein expression. Second, the treatment length was only of 3 months and therefore the identification of biological pathways affected by CR starting early in life, and how these pathways are related to lifespan, cannot be determined. Finally, our findings are exploratory and therefore it is not possible to make causal conclusions about the effects of early onset CR on muscle health. We did not perform any functional measures of skeletal muscle function, such as grip strength. Some studies in CR-animals suggest that this intervention can enhance grip strength in both young (8 months) and old rats (37 months) [43,44]. However, a recent report shows that CR may increase relative grip strength during the first months because of the reduced body weight, although there were no differences in older animals [45]. On the other hand, using the same rat model, our group has already described that energy expenditure and energy efficiency decreased in the first weeks of CR, but became similar to the control group from the fifth week of intervention [16]. It would have also been of interest to check the effect of CR on muscle fiber type in early life, although the collection method was not adequate to perform any histological analyses. In this regard, other authors have shown that CR in older rats prevents fiber loss [46] and does not change fiber type composition, although CR preserved the mitochondrial morphology only in oxidative fibers [18]. Despite these limitations, our study provides convincing evidence that CR started early in life influences some key genes/proteins involved in aging and importantly, these effects are largely sex-dependent.

## 5. Conclusions

In summary, our results suggest a sexual dimorphism in markers of oxidative stress, inflammation, apoptosis, and mitochondrial function in response to CR in young rats. Furthermore, our data show that young female CR treated rats exhibit similar expression patterns of key hallmarks of muscle aging to those observed in older animals under CR, which are related to improved helthspan and lifespan (prolongevity effects). Interestingly, these effects are reduced in younger male CR treated rats. A ‘ceiling effect’ for CR may be present in younger animals, although no adverse effects were reported in skeletal muscle. Future studies are needed to understand whether and how early or late life CR may exert positive effects on healthspan and lifespan in multiple tissues.

## Figures and Tables

**Table 1:** Body and tissue weights, glucose and triglycerides levels in control and restricted rats. Two-way ANOVA was performed to assess for significance. Abbreviations: S, sex differences; D, diet differences; SxD, interactive effect between sex and diet; NS, non-significant. WAT weight: represents sum of inguinal, lumbar, mesenteric, epididymal/ovarian adipose tissues weights. * Statistical differences between calorie restricted and control groups.

	Female			Male			
	ad libitum	Restricted	*p*-Value	ad libitum	Restricted	*p*-Value	Statistics
Body mass (g)	264 ± 6	192 ± 5 *	<0.001	484 ± 13	322 ± 8 *	0.001	S, D, SxD
Liver weight (g)	8.21 ± 0.24	5.64 ± 0.17 *	<0.001	14.7 ± 0.5	9.77 ± 0.29 *	0.005	S, D, SxD
Gastrocnemius weight (g)	3.09 ± 0.08	2.52 ± 0.05 *	<0.001	5.21 ± 0.18	4.26 ± 0.12 *	0.021	S, D
WAT weight (g)	12.7 ± 1.5	4.81 ± 0.5 *	<0.001	30.4 ± 2.4	11.1 ± 0.8 *	<0.001	S, D, SxD
Muscle Somatic Index ×100 (A.u.)	11.7 ± 0.2	13.1 ± 0.3 *	<0.001	11.2 ± 0.1	12.9 ± 0.2 *	0.001	D
Glucose (mg/dL)	153 ± 14	142 ± 7	0.222	176 ± 7	161 ± 9	0.272	NS
Triglycerides (mg/dL)	256 ± 85	182 ± 10	0.183	330 ± 52	179 ± 17	0.024 *	NS

**Table 2:** Protein levels of OXPHOS complexes and mitochondrial fission proteins analyzed by Western Blot. Two-way ANOVA was performed to assess for significance. Abbreviations: S, sex differences; D, diet differences; N, non-significant. * Statistical differences between calorie restricted and control groups.

	Female			Male			
	ad libitum	Restricted	*p*-Value	ad libitum	Restricted	*p*-Value	Statistics
Complex I subunuit NDUFB8 (A.u.)	1.00 ± 0.13	1.06 ± 0.05	0.35	0.59 ± 0.28	0.93 ± 0.19	0.17	NS
Complex II 30 kDa subunit (A.u.)	1.00 ± 0.17	1.44 ± 0.17	0.04 *	0.99 ± 0.40	0.94 ± 0.13	0.45	NS
Complex III, Core protein 2 (A.u.)	1.00 ± 0.26	1.64 ± 0.20	0.04 *	2.13 ± 0.03	1.02 ± 0.26	0.02 *	SxD
Complex IV subunit I (A.u.)	1.00 ± 0.37	2.11 ± 0.65	0.08	1.74 ± 1.01	2.10 ± 0.48	0.37	NS
Complex V alpha subunit (A.u.)	1.00 ± 0.10	1.25 ± 0.09	0.04 *	1.15 ± 0.38	1.07 ± 0.12	0.41	NS
Complex IV/Complex V ratio	1.76 ± 0.59	3.30 ± 1.16	0.14	1.97 ± 1.02	3.32 ± 0.85	0.19	NS
DRP1 (A.u.)	1.00 ± 0.09	1.23 ± 0.18	0.14	1.69 ± 0.37	0.56 ± 0.03	0.01 *	D, SxD
FIS1 (A.u.)	1.00 ± 0.18	1.17 ± 0.20	0.26	1.61 ± 0.40	0.79 ± 0.13	0.02 *	NS

**Table 3:** mRNA expression levels of antioxidant genes. Two-way ANOVA was performed to assess for significance. NS: non-significant. *Sod1*: superoxide dismutase 1; *Sod2*: superoxide dismutase 2; *sirt3*: sirtuin 3; *Nfe2l2*: nuclear factor erythroid 2-related factor 2; *Foxo3*: forkhead box O3.

	Female		Male		
	Restricted vs. ad libitum (Fold-Change)	*p*-Value	Restricted vs. ad libitum (Fold-Change)	*p*-Value	Statistics
*Sod1*	−0.15 ± 0.28	0.26	0.25 ± 0.22	0.35	NS
*Sod2*	−0.48 ± 0.32	0.15	0.30 ± 0.19	0.20	NS
*Sirt3*	−0.21 ± 0.13	0.15	−0.16 ± 0.04	0.17	NS
*Nfe2l2*	0.19 ± 0.11	0.18	−0.10 ± 0.17	0.40	NS
*Foxo3*	0.10 ± 0.21	0.38	0.27 ± 0.08	0.05	NS

**Table 4:** Protein levels of antioxidant enzymes and factors analyzed by Western Blot. Two-way ANOVA was performed to assess for significance. Abbreviations: S, sex differences; NS, non-significant. * Statistical differences between calorie restricted and control groups.

	Female			Male			
	ad libitum	Restricted	*p*-Value	ad libitum	Restricted	*p*-Value	Statistics
SOD 2 (A.u.)	1.00 ± 0.29	0.96 ± 0.20	0.46	1.44 ± 0.68	1.13 ± 0.59	0.38	NS
Acetylated SOD2 (A.u.)	1.00 ± 0.18	1.33 ± 0.20	0.12	1.43 ± 0.21	1.22 ± 0.21	0.28	NS
Acetylated SOD2/Total SOD2 ratio	9.3 ± 1.0	11 ± 1.1	0.11	9.4 ± 3.2	12 ± 3.4	0.31	NS
SIRT3 (A.u.)	1.00 ± 0.19	2.46 ± 0.42	0.01 *	1.33 ± 0.08	1.60 ± 0.34	0.31	NS
NRF2 (A.u.)	1.00 ± 0.10	0.93 ± 0.07	0.27	0.48 ± 0.27	0.90 ± 0.15	0.10	S
FOXO3A (A.u.)	1.00 ± 0.36	0.93 ± 0.38	0.45	0.43 ± 0.27	0.72 ± 0.22	0.23	NS

**Table 5:** mRNA expression levels of autophagy markers. Two-way ANOVA was performed to assess for significance. Abbreviations: NS, non-significant; *Map1lc3a*, microtubule-associated protein 1 light chain 3 alpha; *Sqstm1*: sequestosome 1.

	Female		Male		
	Restricted vs. ad libitum (Fold-Change)	*p*-Value	Restricted vs. ad libitum (Fold-Change)	*p*-Value	Statistics
*Map1lc3a*	0.89 ± 0.25	0.13	0.34 ± 0.23	0.15	NS
*Sqstm1*	−0.07 ± 0.13	0.36	0.26 ± 0.08	0.06	NS

**Table 6:** Protein levels of LC3-I and LC3-II, and caspase 3 analyzed by Western Blot. Two-way ANOVA was performed to assess for significance. Abbreviations: S, sex differences; D, diet differences; SxD, interactive effect between sex and diet; NS, non-significant.

	Female			Male			
	ad libitum	Restricted	*p*-Value	ad libitum	Restricted	*p*-Value	Statistics
LC3 I (A.u.)	1.00 ± 0.13	1.19 ± 0.12	0.16	0.59 ± 0.10	0.82 ± 0.08	0.07	NS
LC3 II (A.u.)	1.00 ± 0.16	1.35 ± 0.18	0.08	0.85 ± 0.20	1.09 ± 0.20	0.22	NS
LC3-II/LC3-I ratio	0.34 ± 0.03	0.4 ± 0.05	0.14	0.49 ± 0.05	0.44 ± 0.04	0.24	NS
Caspase 3 (A.u.)	1.00 ± 0.15	1.00 ± 0.09	0.49	1.11 ± 0.30	0.34 ± 0.11	0.07	S, D, SxD

**Table 7:** mRNA expression levels of key autophagy markers. Two-way ANOVA was performed to assess for significance. S indicates sex effect; SxD indicates interactive effect between sex and diet. NS: non-significant; * Significant differences between calorie-restricted and control groups. *Pik3ca*: phosphatidylinositol-4,5-bisphosphate 3-kinase, catalytic subunit alpha; *Akt1*: AKT serine/threonine kinase 1; *Gsk3b*: glycogen synthase kinase 3 beta; *Mtor*: mechanistic target of rapamycin kinase; *Hif1a*: hypoxia inducible factor 1 subunit alpha.

	Female		Male		
	Restricted vs. ad libitum (Fold-Change)	*p*-Value	Restricted vs. ad libitum (Fold-Change)	*p*-Value	Statistics
*Pik3ca*	0.41 ± 0.21	0.08	0.01 ± 0.22	0.49	NS
*Akt1*	0.44 ± 0.42	0.22	−0.69 ± 0.43	0.12	NS
*Gsk3b*	0.10 ± 0.24	0.36	−0.23 ± 0.13	0.16	NS
*mTOR*	0.79 ± 0.57	0.15	0.04 ± 0.29	0.46	NS
*Hif1a*	−0.05 ± 0.25	0.45	−0.49 ± 0.06	<0.01 *	S, SxD

**Table 8:** Protein levels of several nutrient-sensing pathways markers analyzed by Western Blot. Two-way ANOVA was performed to assess for significance. D indicates diet differences. NS: non-significant. * Statistical differences between calorie restricted and control groups. All data were normalized to ad libitum females.

	Female			Male			
	ad libitum	Restricted	*p*-Value	ad libitum	Restricted	*p*-Value	Statistics
IRb (A.u.)	1.00 ± 0.27	1.22 ± 0.20	0.26	0.74 ± 0.36	0.63 ± 0.09	0.38	NS
IRS1 (A.u.)	1.00 ± 0.39	0.53 ± 0.10	0.16	0.64 ± 0.29	0.79 ± 0.18	0.33	NS
AMPK (A.u.)	1.00 ± 0.12	0.76 ± 0.08	0.07	1.03 ± 0.13	0.56 ± 0.34	0.19	NS
LKB1 (A.u.)	1.00 ± 0.14	0.79 ± 0.11	0.14	1.07 ± 0.06	0.90 ± 0.33	0.37	NS
AKT (A.u.)	1.00 ± 0.15	0.90 ± 0.08	0.28	1.10 ± 0.21	1.01 ± 0.19	0.28	NS
GSK (A.u.)	1.00 ± 0.24	0.51 ± 0.15	0.05	0.36 ± 0.09	0.50 ± 0.02	0.10	NS
ERRA (A.u.)	1.00 ± 0.26	1.21 ± 0.21	0.27	0.75 ± 0.31	1.17 ± 0.36	0.21	D
LDHA (A.u.)	1.00 ± 0.16	0.75 ± 0.10	0.10	0.77 ± 0.01	0.49 ± 0.12	0.10	NS
IDH2 (A.u.)	1.00 ± 0.25	0.79 ± 0.12	0.23	0.25 ± 0.03	0.61 ± 0.11	0.04 *	NS

**Table 9:** mRNA expression levels of inflammatory markers. Two-way ANOVA was performed to assess for significance. NS: non-significant. * Statistical differences between calorie restricted and control groups. Rela: RELA proto-oncogen, nuclear factor kappa B subunit; Il1b: interleukin 1 beta; Tgfb1: transforming growth factor beta 1; Tnf: tumor necrosis factor.

	Female		Male		
	Restricted vs. ad libitum (Fold-Change)	*p*-Value	Restricted vs. ad libitum (Fold-Change)	*p*-Value	Statistics
*Rela*	0.20 ± 0.12	0.25	0.05 ± 0.09	0.25	NS
*Il1b*	−1.20 ± 0.50	0.16	0.24 ± 0.28	0.04 *	NS
*Tgfb1*	0.49 ± 0.35	0.15	−0.63 ± 0.69	0.26	NS
*Tnf*	−0.17 ± 0.54	0.39	−0.23 ± 0.51	0.36	NS

**Table 10:** Protein levels of several inflammation-related pathways markers analyzed by Western Blot. Two-way ANOVA was performed to assess for significance. NS: non-significant. * Statistical differences between calorie restricted and control groups. All data were normalized to ad libitum females.

	Female			Male			
	ad libitum	Restricted	*p*-Value	ad libitum	Restricted	*p*-Value	Statistics
IL1R (A.u.)	1.00 ± 0.16	1.13 ± 0.20	0.30	0.52 ± 0.04	0.89 ± 0.28	0.17	NS
IL6 (A.u.)	1.00 ± 0.10	0.67 ± 0.08	0.01 *	0.66 ± 0.12	0.87 ± 0.29	0.29	NS
NF-κB (A.u.)	1.00 ± 0.13	1.42 ± 0.20	0.04 *	0.55 ± 0.19	0.37 ± 0.01	0.15	NS
IκB (A.u.)	1.00 ± 0.16	0.97 ± 0.13	0.45	1.09 ± 0.02	0.68 ± 0.12	0.04 *	NS

**Table 11:** mRNA expression levels of senescence markers. Two-way ANOVA was performed to assess for significance. NS: non-significant. *Cdkn1a*: cyclin-dependent kinase inhibitor 1A; *Cdkn2a*: cyclin-dependent kinase inhibitor 2A; *Tp53*: tumor protein p53; *Sirt6*: sirtuin 6.

	Female		Male		
	Restricted vs. ad libitum (Fold-Change)	*p*-Value	Restricted vs. ad libitum (Fold-Change)	*p*-Value	Statistics
*Cdkn1a*	0.14 ± 0.26	0.43	1.28 ± 0.37	0.07	NS
*Cdkn2a*	1.11 ± 0.62	0.09	−0.50 ± 0.44	0.21	NS
*Tp53*	0.74 ± 0.54	0.12	−0.56 ± 0.38	0.17	NS
*Sirt6*	0.78 ± 0.07	0.20	−0.43 ± 0.28	0.14	NS

## Data Availability

The data used to support the findings of this study are available from the corresponding author upon request.

## Supplementary Materials

The following supporting information can be downloaded at: https://www.mdpi.com/article/10.3390/nu14214535/s1; Supplemental Table S1. Antibodies used for Western Blot. Supplemental Table S2. Primers and conditions used for RT-qPCR.
