# Supplementary material for: Sex Specific Differences in Response to Calorie Restriction in Skeletal Muscle of Young Rats
Source: Nutrients. 2022 Oct 28;14(21):4535. doi: 10.3390/nu14214535 (PMC9658986; doi:10.3390/nu14214535)

Supplementary Materials

Supplemental Table S1. Antibodies used for Western Blot.

Target	Dilution	Source	Target	Dilution	Source
Total OXPHOS Rodent Cocktail	1:500	Abcam (ab110413)	SIRT3	1:1000	Cell Signaling (cs2627)
DRP1	1:1000	Santa Cruz (sc-32898)	FOXO3A	1:1000	Cell Signaling (cs2497)
FIS1	1:500	Santa Cruz (sc-98900)	NRF2	1:1000	Abcam (ab89443)
SOD2	1:500	Santa Cruz (sc-30080)	LC3A/B	1:1000	Cell Signaling (cs4108)
Acetylated SOD2 (K68)	1:1000	Abcam (ab137037)	IRb	1:1000	BD Biosciences (#610109)
ERRA	1:1000	Cell Signaling (cs13826)	IRS1	1:500	Cell Signaling (cs3407)
RIL1	1:1000	Abcam (AB40774)	AKT	1:1000	Cell Signaling (cs9272)
IL6	1:1000	Santa Cruz (sc-57315)	AMPK	1:1000	Cell Signaling (cs2522)
NFkB	1:500	Santa Cruz (sc-372)	LKB1	1:1000	Cell Signaling (cs13031)
IkB	1:1000	Cell signaling (cs4814)	LDHA	1:1000	Cell Signaling (cs2021)
P53	1:500	Santa Cruz (sc-263)	GSK3	1:500	Santa Cruz (sc-9166)
Caspase 3	1:1000	Cell Signaling (cs9662)	IDH2	1:1000	Cell Signaling (cs12652)

Supplemental Table S2. Primers and conditions used for RT-qPCR.

Gene	Forward Primer (5′-3′)<br>Reverse Primer (5′-3′)	An. T (°C)	Gene	Forward Primer (5′-3′)<br>Reverse Primer (5′-3′)	An. T (°C)
Rpl32	CCAGTCGGACCGATATGTGAA<br>TCTGGCCCTTGAATCTTCTCC	60	*Gsk3b*	AAGGCACATCCTTGGACGAA<br>GTTGAAGAGGGCAGGTGTGT	58
Tbp	CACCGTGAATCTTGGCTGTAAAC<br>CGCAGTTGTTCGTGGCTCTC	60	*Mtor*	CTGATGTCATTTATTGGCACAAA<br>CAGGGACTCAGAACACAAATGC	57
Sod1	ACTTCGAGCAGAAGGCAAGC<br>CCAGGTCTCCAACATGCCTC	60	*Hif1a*	GCGGCGAGAACGAGAAGAA<br>AGATGGGAGCTCACGTTGTG	60
Sod2	ACCGAGGAGAAGTACCACGA<br>TAGGGCTCAGGTTTGTCCAG	60	*Rela*	GAACTTGTGGGGAAGGACTG<br>GGGGTTATTGTTGGTCTGGA	60
Sirt3	AGGCCCATATCCCTCTCTGT<br>ACTCCCTGGGGATCTGAAGT	60	Il1b	CTGTGACTCGTGGGATGATG<br>GGGATTTTGTCGTTGCTTGT	60
Nfe2l2	GCAACTCCAGAAGGAACAGG<br>AGGCATCTTGTTTGGGAATG	58	Tgfb1	GCAACAACGCAATCTATGAC<br>CCTGTATTCCGTCTCCTT	60
Foxo3	TTCGTTCTGAACCCGCATGA<br>CGGCTCACTTTGTCCCAGAT	60	Tnf	CTGAACTTCGGGGTGATCGG<br>CTTGGTGGTTTGCTACGACG	60
Map1lc3a	GGTCCAGTTGTGCCTTTATTGA<br>GTGTGTGGGTTGTGTACGTCG	60	Cdkn1a	GAGCAGTGCCCGAGTTAAGG<br>TGGAACAGGTCGGACATCAC	60
Sqstm1	CTAGGCATCGAGGTTGACATT<br>CTTGGCTGAGTACCACTCTTATC	56	Cdkn2a	TCCTCCGCTGGGAACGT<br>GGCGTGCTTGAGCAGAAGTT	55
Pik3ca	ACCTCAGGCTTGAAGAGTGTCG<br>CCGTAAGTCGTCGCCATTTTTA	59	Tp53	TTCCCTCAATAAGCTGTTCTGCC<br>TGCTCTCTTTGCACTCCCTGG	66
Akt1	CTAACTTGAGCCGCAGGAAC<br>GCTTGCTCAGTTTGCTACCC	57	Sirt6	GACCTAACGCTCGCTGATGA<br>CTGGCGGTCATGTTTTGTG	60

An. T: annealing temperature; *Rpl32*: ribosomal protein L32; *Tbp*: TATA box binding protein; *Sod1*: superoxide dismutase 1; *Sod2*: superoxide dismutase 2; *sirt3*: sirtuin 3; *Nfe2l2*: nuclear factor erythroid 2-related factor 2; *Foxo3*: forkhead box O3; *Map1lc3a*: microtubule-associated protein 1 light chain 3 alpha; *Sqstm1*: sequestosome 1; *Pik3ca*: phosphatidylinositol-4,5-bisphosphate 3-kinase, catalytic subunit alpha; *Akt1*: AKT serine/threonine kinase 1; *Gsk3b*: glycogen synthase kinase 3 beta; *Mtor*: mechanistic target of rapamycin kinase; *Hif1a*: hypoxia inducible factor 1 subunit alpha; *Rela*: RELA proto-oncogen, nuclear factor kappa B subunit; *Il1b*: interleukin 1 beta; *Tgfb1*: transforming growth factor beta 1; *Tnf*: tumor necrosis factor; *Cdkn1a*: cyclin-dependent kinase inhibitor 1A; *Cdkn2a*: cyclin-dependent kinase inhibitor 2A; *Tp53*: tumor protein p53; *Sirt6*: sirtuin 6.

Figure S1. ATPase activity as a measure of muscle function. Effects of calorie restriction on the enzymatic activity of Complex V. Results are shown as the mean ± SEM. Two-way ANOVA was performed to assess for significance. Abbreviations: S, sex differences, p=0.00215.

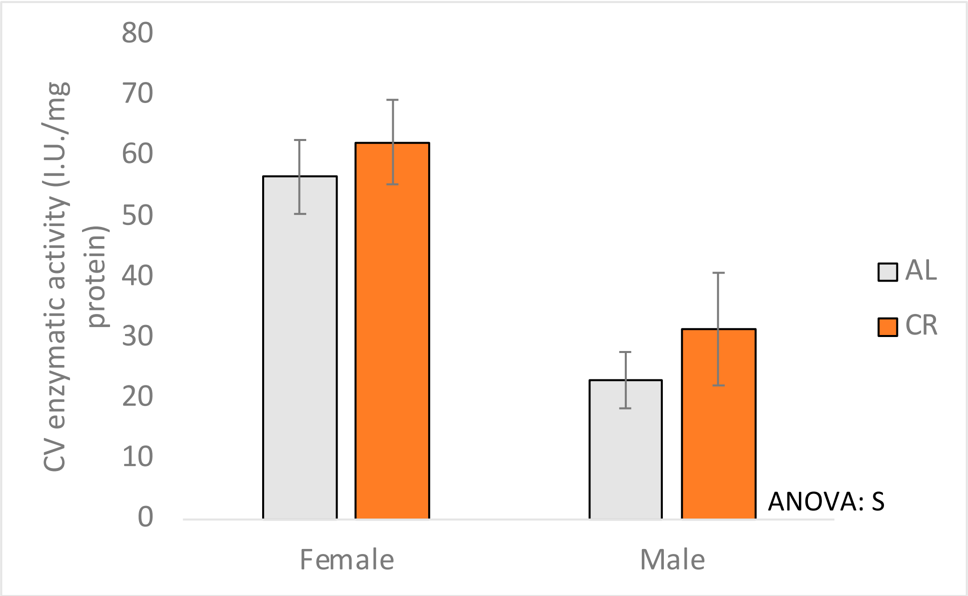

Supplement: Supplementary file 1 [file nutrients-14-04535-s001.zip › nutrients-1928874-supplementary.pdf]
